# Soziale Roboter im Schweizer Gesundheitswesen – Einsatzmöglichkeiten, Chancen und Risiken aus der Sicht von potenziellen Anwendergruppen

**DOI:** 10.1007/s11612-022-00646-5

**Published:** 2022-09-22

**Authors:** Iris Kramer, Nicole Zigan, Alexandra Tanner, Hartmut Schulze, Maria Schubert

**Affiliations:** 1 Institut für Pflege, ZHAW Zürcher Hochschule für Angewandte Wissenschaften, Winterthur, Schweiz; 2 Hochschule für Angewandte Psychologie, Fachhochschule Nordwestschweiz FHNW, Olten, Schweiz

**Keywords:** Soziale Roboter, Gesundheitswesen, Gesundheitspersonal, Mensch-Roboter-Interaktion, Nutzerperspektiven, Social robot, Healthcare, Healthcare professionals, Human-robot-interaction, User perspectives

## Abstract

Dieser Beitrag der Zeitschrift Gruppe. Interaktion. Organisation. (GIO) berichtet und vertieft die Ergebnisse eines interprofessionellen Projektes im Auftrag der TA-SWISS (Stiftung für Technologiefolgen-Abschätzung). In dem Projekt wurde u. a. untersucht, wie soziale Roboter aus der Sicht von Gesundheitsfachpersonen und von Bewohnerinnen und Bewohnern eines Alterszentrums zukünftig in der Schweiz eingesetzt werden können und welche Chancen und Risiken sie bei diesem Einsatz sehen. Hintergrund dieser Fragestellung war, dass soziale Roboter zunehmend im Gesundheitsbereich eingesetzt werden, die Perspektiven der potenziellen Anwenderinnen und Anwender jedoch nur wenig bekannt sind. Daher wurde mit einem explorativen Studiendesign im August 2020 ein Workshop („Round Table Gesundheit“) mit 26 Teilnehmenden durchgeführt, der Roboterszenarien, vier Fokusgruppeninterviews und eine Nachbefragung beinhaltete. Es zeigte sich in der vertieften Analyse, dass sich die von den Teilnehmenden genannten Einsatzmöglichkeiten von sozialen Robotern einerseits in zwei Anwendergruppen („Patientinnen/Patienten“ und „Gesundheitsfachpersonen“) und andererseits in zwei Rollen des sozialen Roboters (persönlich zugeordnet – „persönlicher Buddy/Assistent“ und nicht persönlich zugeordnet – „hilfreicher Kollege“) einteilen liessen. Die Chancen und Risiken sozialer Roboter liessen sich in die drei Spannungsfelder „Selbstbestimmung vs. soziale Isolation“, „Entlastung vs. Belastung & Abhängigkeit“ und „Sicherheit vs. Gefahren“ kategorisieren. Von potenziellen Anwenderinnen und Anwendern werden somit vielfältige Einsatzmöglichkeiten und Chancen für soziale Roboter im Gesundheitsbereich gesehen. Gleichzeitig gilt es auch die Risiken zu berücksichtigen und zu minimieren, damit soziale Roboter zukünftig erfolgreich in der Praxis eingesetzt werden können.

## Abstract (en)

This article from the journal Gruppe. Interaktion. Organisation. (GIO) offers an in-depth analysis and discussion of the results of an inter-professional project on behalf of TA-SWISS (Foundation for Technology Assessment). The project investigated how social robots could be used in the future in Switzerland from the perspective of health professionals and elderly residents and what opportunities and risks they see in this use. The background of this question was that social robots are increasingly used in the healthcare sector while the perspectives of potential users are hardly known. Therefore, an exploratory study design was used to conduct a workshop (“Round Table Health”) with 26 participants in August 2020, which included robot scenarios, four focus group interviews and a follow-up survey. The in-depth analysis showed that the possible uses of social robots mentioned by the participants could not only be divided into two user groups (“patients” and “health professionals”) but also into two roles of the social robot (personally assigned—“personal buddy/assistant” and not personally assigned—“helpful colleague”). The opportunities and risks of social robots could be categorised into the three areas of tension, i.e. “self-determination vs. social isolation”, “relief vs. burden & dependence” and “safety vs. dangers”. In conclusion, potential users see a wide range of possible uses and opportunities for social robots in the health sector, yet it is important to consider and minimise the risks so that social robots can be successfully used in practice in the future.

## Einleitung

Durch die immer ausgereiftere Robotiktechnologie steigt auch im europäischen Raum zunehmend das Interesse, Roboter ausserhalb der Industrie in sozialen Bereichen, z. B. in der Bildung (z. B. in Schulen), in öffentlichen Bereichen (z. B. in Museen oder Geschäften) oder im privaten Bereich (Zuhause) einzusetzen (Bendel 2021a). Im Rahmen der Digitalisierung im Gesundheitswesen ist der Einsatz von nicht-sozial assistiven Robotern, wie Operationsroboter (z. B. Da Vinci) oder Roboter für physische Therapie (z. B. Lokomat) von Gesundheitsfachpersonen und der Bevölkerung bereits akzeptiert (Khan et al. 2020). Hingegen ist der Einsatz von sozial assistiven Robotern, oder kurz sozialen Robotern, wie Service- und Begleitroboter (gemäß Busse et al. 2021), in den letzten Jahren zunehmend zu beobachten. Allerdings findet dies bisher vor allem in Form von Pilotstudien statt (Schulze et al. 2021).

Treiber dieser zunehmenden Einsätze sozialer Robotik im Gesundheitsbereich sind unter anderem die älter werdende Bevölkerung, ein persistenter und zunehmender Mangel an Gesundheitsfachpersonen sowie die aktuelle COVID-19-Pandemie (Deloitte 2020). Aufgrund des zunehmenden hohen Alters, das mit einem Anstieg von mehrfach chronischen Erkrankungen verbunden ist, gehen die Prognosen von einem steigenden Unterstützungsbedarf in alltäglichen Aktivitäten aus (Bundesamt für Gesundheit (BAG) 2021a). Gleichzeitig besteht in der Bevölkerung der Wunsch nach Autonomie und sozialer Teilhabe, auch im sehr hohen Alter (BAG 2019). Zusätzlich ist von einer sinkenden Anzahl an Ressourcen/Kapazitäten für diese Pflege und Betreuung auszugehen (Deloitte 2020). Bekannte Gründe hierfür sind der zunehmende Mangel an qualifiziertem Gesundheitspersonal (Merçay et al. 2021), die unattraktiven Arbeitsbedingungen sowie die steigende Belastung des Gesundheitspersonals (BAG 2021b; Peter et al. 2021), welche sich unter der aktuellen COVID-19-Pandemie noch verschärft haben (Riguzzi et al. 2021).

Die COVID-19-Pandemie wirkte sich als Beschleuniger von Einsätzen verschiedenartiger Roboter aus, dies trifft auch für die Anwendung von sozialen Robotern zu (Khan et al. 2020). Erst kürzlich hielten Henschel et al. (2021) in ihrem Review fest, dass in der Literatur zur sozialen Robotik keine allgemein anerkannte Definition für Roboter existiert. Darüber hinaus fehle ein Konsens darüber, was das „Soziale“ an sozialen Robotern ausmacht. Gemäß Henschel et al. (2021) nehmen soziale Roboter im Bereich der Human-Robot-Interaktion eine besondere Rolle ein und lassen sich in die Kategorie der nahen Interaktion einordnen, bei der Menschen und Roboter zumindest potenziell als Gleichgestellte oder Gefährten interagieren. Bendel (2021b) beschreibt in einem ähnlichen Verständnis, welches für diesen Beitrag leitend ist, soziale Roboter als

[…] sensomotorische Maschinen, die für den Umgang mit Menschen oder Tieren geschaffen wurden. Sie können über fünf Dimensionen bestimmt werden, nämlich die Interaktion mit Lebewesen, die Kommunikation mit Lebewesen, die Nähe zu Lebewesen, die Abbildung von (Aspekten von) Lebewesen sowie – im Zentrum – den Nutzen für Lebewesen.

Als bekannte Beispiele für soziale Roboter können Paro oder Nao genannt werden. Durch die unterschiedliche äussere Gestalt (z. B. tierähnlich oder humanoid) und die Auswahl an eingebauten technischen Funktionalitäten (z. B. Sensoren, Kameras) sind soziale Roboter in der Lage, mit Menschen zu interagieren: sie können z. B. kommunizieren, auf Emotionen reagieren, zu verschiedenen Aktivitäten motivieren und nehmen so unterschiedliche Rollen in der Mensch-Roboter-Interaktion ein (Onnasch und Roesler 2021). Soziale Roboter können pflegebedürftige Menschen im Alltag unterstützen und die soziale Teilhabe fördern (Zöllick et al. 2021). Aktuell werden Möglichkeiten und Effekte eines Einsatzes von sozialen Robotern v. a. im pädiatrischen und insbesondere im gerontologischen Bereich untersucht, z. B. in Pflegeheimen oder bei Menschen mit Demenz zur körperlichen Assistenz oder zur kognitiven und emotionalen Unterstützung (Sheridan 2020; Koh et al. 2021; Littler et al. 2021; Alabdulkareem et al. 2022). Auch für die psychische Gesundheit wurden Einsätze von sozialen Robotern geprüft und es wurden im Allgemeinen positive Effekte gefunden, allerdings handelt es sich hier überwiegend um Studien mit eingeschränkter Aussagekraft (Guemghar et al. 2022).

Trotz zunehmendem Einsatz von sozialen Robotern haben jedoch bisher nur wenige Personen im Gesundheitsbereich bereits Erfahrungen mit sozialen Robotern sammeln können (Seibert et al. 2020). Die Vorstellungen sind daher häufig noch von Kinofilmen und Science-Fiction geprägt. Damit soziale Roboter bedarfsgerecht entwickelt und in der Praxis wirksam eingesetzt werden können, ist es zentral, die Perspektiven von potenziellen Anwenderinnen und Anwendern frühzeitig einzubeziehen (Papadopoulos et al. 2020).

Um diese Perspektiven genauer zu untersuchen, wurde im Auftrag der TA-SWISS (Stiftung für Technologiefolgen-Abschätzung) in der Schweiz im Zeitraum von 2019–2021 ein interdisziplinäres Projekt zur Technologiefolgenabschätzung sozialer Roboter in verschiedenen Anwendungsfeldern, unter anderem im Gesundheitsbereich, durchgeführt. In diesem Projekt wurden die Einsatzmöglichkeiten, Chancen und Risiken sozialer Roboter in Gesundheitsinstitutionen in der Schweiz untersucht (Schulze et al. 2021).

Ziel des vorliegenden Beitrages ist es, die Ergebnisse dieses Projektes zum Bereich Gesundheit mittels einer vertieften und ergänzenden Analyse weiter zu explorieren und zu diskutieren. Folgende Fragen sind hierbei leitend:

1. Wie beschreiben und gewichten potenzielle Anwenderinnen und Anwender die Einsatzmöglichkeiten von sozialen Robotern im Anwendungsfeld Gesundheit?
2. Welche übergeordneten Spannungsfelder liegen den von den potenziellen Anwenderinnen und Anwendern genannten Chancen und Risiken sozialer Roboter im Anwendungsfeld Gesundheit zugrunde?

## Methode

Für das Projekt wurde aufgrund des limitierten Forschungsstandes zur Perspektive von potenziellen Anwendergruppen von sozialen Robotern in der Schweiz ein exploratives Design mit einer Methodentriangulation gewählt. Diese Methode umfasste einen Erhebungshalbtag, den wir für eine vereinfachte Aussenkommunikation mit den Teilnehmenden „Round Table Gesundheit“ genannt haben. Der Round Table beinhaltete vier qualitative Fokusgruppeninterviews, um die Perspektiven der Teilnehmenden zu explorieren und vertieft zu verstehen. Zudem wurde im Nachgang an den Round Table eine quantitative Online-Nachbefragung eingesetzt, um die Aussagen aus den Fokusgruppeninterviews zu ergänzen, zu verifizieren und zu gewichten.

### Teilnehmende und Rekrutierung

Es erfolgte eine zielgerichtete Rekrutierung von Personen, die im stationären und ambulanten Gesundheitsbereich tätig oder involviert waren und welche voraussichtlich in Zukunft vermehrt mit sozialen Robotern interagieren.

### Datensammlung

Der Round Table wurde im August 2020 in einem hybriden Format, also an zwei digital vernetzten Orten in der Deutschschweiz, einer Fachhochschule und einem Alters‑/Pflegezentrum, durchgeführt. Die Aufteilung der Teilnehmenden an diese zwei Orte war aufgrund der COVID-19-Pandemie und den zum aktuellen Zeitpunkt geltenden Sicherheitsbestimmungen erforderlich.

#### Ablauf Round Table

Als Einstieg in den Round Table erlebten die Teilnehmenden zunächst vier Roboterszenarien, davon drei 15-minütige Roboter-Live-Szenarien und ein videobasiertes Szenario. Das Ziel der Roboterszenarien war es, ein gemeinsames Verständnis von sozialen Robotern für die Teilnehmenden als Diskussionsgrundlage zu den Fokusgruppeninterviews zu schaffen. Für die Szenarien und Fokusgruppeninterviews wurden die Teilnehmenden in vier Gruppen à fünf bis sieben Personen eingeteilt. Die Gruppeneinteilung und die Roboterszenarien können der Tab. 1 entnommen werden.

**Table 1:** 

*Gruppe A interdisziplinär*	1 Ärztin, 2 IT-Spezialisten, 1 Leiterin Sozialdienst, 2 Therapeuten, 1 Managementperson
*Gruppe B Management*	5 Managementpersonen, z. B. Pflegedienstleitung, Leitung Betreuung, Stv. Direktion Patientenvertretungsorganisation
*Gruppe C Pflege*	7 Pflegefachpersonen
*Gruppe D Langzeitbereich*	4 Bewohnende, 1 Managementperson, 1 Pflegefachperson, 1 IT-Spezialist
*Roboterszenario 1*	Roboter Nao (Avatarion Technology): Aktivierungsübungen: Bewegung und Musik
*Roboterszenario 2*	Roboter Lio (F&P Robotics): Assistenzfunktionen: Becher bringen, Therapie: Gedächtnisübungen
*Roboterszenario 3*	Roboter Pepper (raumCode): direkte Interaktion mit Teilnehmenden mit verschiedenen Sprecharten
*Roboterszenario 4*	Videozusammenschnitte anderer sozialer Roboter (z. B. Paro)

#### Fokusgruppeninterviews

Die Fokusgruppeninterviews basierten auf einem durch Literatur und Expertenwissen gestützten Leitfaden und dauerten ca. eine Stunde. Dabei wurden Einsatzmöglichkeiten sozialer Roboter für Patientinnen und Patienten in Spitälern, Klientinnen und Klienten zuhause sowie Bewohnerinnen und Bewohner in Alters- und Pflegezentren aber auch für das Gesundheitspersonal sowie die damit verbundenen Chancen und Risiken thematisiert. Die Interviews wurden mit einem Audiogerät aufgenommen, gleichzeitig wurden die Kernaussagen mittels Knowledge Mapping, einer ressourcenschonenden, gleichzeitigen Datenerhebungs‑, Analyse- und Präsentationsmethode (Pelz et al. 2004), auf Papier festgehalten. Nach den Interviews wurden die sogenannten Knowledge Maps im Plenum den Teilnehmenden aus allen Fokusgruppen vorgestellt, somit fand eine konsensuale Validierung über die Vollständigkeit der Knowledge Maps statt (Pelz et al. 2004).

#### Nachbefragung

Der Fragebogen für die einmalige Online-Nachbefragung wurde projektspezifisch im Vorfeld des Round Tables auf Basis einer Literaturanalyse und der erwarteten Ergebnisse des Round Tables von der Projektgruppe entwickelt und inhaltlich verifiziert. In der Literaturanalyse zeigte sich, dass soziale Roboter hauptsächlich bei älteren Menschen und Kindern/Jugendlichen in den Rollen des Assistenten für funktionale Aufgaben (z. B. einen Gegenstand holen), als Manager/Therapeut bei kognitiven Aufgaben (z. B. an Termine erinnern), als Trainer/Wächter im Gesundheitsmanagement (z. B. Monitoring von Vitalzeichen) und als emotionaler Therapeut (z. B. Begleiter oder sozialer Vermittler) eingesetzt wurde (Schubert et al. 2021). An diese Rollen angelehnt wurden die folgenden Rollen im Hauptteil des Fragebogens entwickelt: (1) psychologischer Therapeut z. B. Spiel‑/Gesprächspartner; (2) körperliche Aktivierung, z. B. Gymnastikübungen; (3) pflegerische Aufgaben, z. B. Waschen/Mobilisierung; (4) Dienstleistungen, z. B. holen und bringen von Gegenständen, Telefonzentrale, Reminder. Die entsprechenden Rollen wurden in den drei Altersgruppen Kinder und Jugendliche, Erwachsene und ältere Menschen mit gesundheitlichen Einschränkungen auf einer sechsstufigen Likert-Skala von 1 = „trifft überhaupt nicht zu“ bis 6 = „trifft voll und ganz zu“ bewertet. In den Fokusgruppeninterviews unterschieden sich die Aussagen oft danach, ob der Roboter autonom und unabhängig vom Gesundheitsfachpersonal handelt oder ob der Roboter zusammen mit dem Gesundheitspersonal eingesetzt wird. Deshalb wurden die Aussagen einmal als „eigenständig“ und einmal als „zusammen mit medizinischem Personal“ bewertet. Auch die Chancen und Risiken wurden auf einer sechsstufigen Likert-Skala von 1 = „kein Risiko“/„keine Chance“ bis 6 = „grosses Risiko“/„grosse Chance“ bewertet. Der Fragebogen umfasste zudem demografische Angaben und Fragen zu Einstellungen und Vorwissen zu Robotern inkl. Vorerfahrungen mit sozialen Robotern (selbstentwickelte Items), Technikaffinität (einzelne Items in Anlehnung an Karrer et al. 2009), Akzeptanz sozialer Roboter (einzelne Items in Anlehnung an Heerink et al. 2010) und zukünftiger Nutzungsabsicht sozialer Roboter (einzelne Items in Anlehnung an Xu (2019)). Der Link zum Fragebogen wurde zwei Wochen nach dem Round Table allen Teilnehmenden, ausser den Bewohnenden, per E‑Mail via Questback gesendet.

### Datenanalyse

#### Fokusgruppeninterviews

Die Analyse der Fokusgruppeninterviews folgte der Methode des Knowledge Mappings, die einen zusammenfassenden, inhaltsanalytischen Auswertungsprozess vorsieht und eine schrittweise Verdichtung und Ordnung der Diskussionsinhalte beinhaltet (Pelz et al. 2004). Nach den Interviews führten die Erst- und Zweitautorin eine Qualitätssicherung der Knowledge Maps anhand der Audioaufnahmen durch und vervollständigten sie wo nötig (gemäß Rettke et al. 2015). Anschliessend wurden die Aussagen zu den Einsatzmöglichkeiten, Chancen und Risiken aus den vier Fokusgruppen zusammengetragen und mit den Interviewmoderierenden verifiziert. Weiterhin erfolgte eine Reduktion auf die wesentlichen Kernaussagen und dessen grafische Darstellung in den übergeordneten Knowledge Maps. Für die vertiefte Analyse im vorliegenden Beitrag wurden die Knowledge Maps zu den Chancen und Risiken sozialer Roboter in einer vergleichenden Art und Weise zusammengebracht, damit die gegenseitige Abhängigkeit deutlicher wird. Dem Knowledge Map für die Einsatzmöglichkeiten wurde in der vertieften Analyse eine zweite Dimension hinzugefügt, so dass die Teaming- und Kollaborationsansätze klarer hervortreten konnten.

#### Nachbefragung

Die Analyse der Nachbefragung erfolgte mit deskriptiven Statistiken wie Häufigkeiten und prozentualen Anteile sowie Median (Md) und Interquartilsabstand (IQR). Für die Auswertung der Bewertungen der Chancen und Risiken wurden die Items wie folgt dichotomisiert: Ausprägungen 1 bis 3 = „eher klein“, Ausprägungen 4 bis 6 = „eher gross“. Die Datenanalyse wurde mit dem Statistikprogram IBM SPSS Statistics Version 28 (IBM Corp. 2021) durchgeführt.

### Ethische Überlegungen

Dieses Projekt wurde in Übereinstimmung mit der Deklaration von Helsinki (World Medical Association 2013) und den Guidelines für Gute Klinische Praxis (ICH 2016) durchgeführt. Nach Schweizer Humanforschungsgesetz war eine Bewilligung durch eine Ethikkommission nicht notwendig, da keine gesundheitsbezogenen Daten erhoben wurden. Von allen Round Table-Teilnehmenden wurde eine informierte Einwilligungserklärung eingeholt.

## Ergebnisse

Bei den Teilnehmenden handelte es sich mehrheitlich um Managementpersonen und Pflegepersonen (s. Tab. 1), 58 % der Teilnehmenden waren Frauen und die Teilnehmenden waren zwischen 33 und 93 Jahre, im Durchschnitt 52 Jahre, alt. Von den Teilnehmenden arbeiteten neun Personen in Akutspitälern, drei Personen im psychiatrischen Bereich, acht Personen vertraten Alters‑/Pflegezentren, zwei waren in Patientenvertretungsorganisationen tätig, zwei in Fach- und Beratungsstellen und je eine Person in der Spitex (ambulante/häusliche Pflege) und in einer Fachhochschule.

Detailliertere Beschreibungen der einzelnen Chancen, Risiken und Einsatzmöglichkeiten sind im Abschlussbericht des Projektes (Schulze et al. 2021) zu finden, nachfolgend werden die Ergebnisse der vertiefteren Analyse aus den Fokusgruppeninterviews und der Nachbefragung dargestellt. Dabei wird zunächst die Fragestellung nach den genannten Einsatzmöglichkeiten und deren Gewichtung beantwortet und danach die Fragestellung zu den übergeordneten Spannungsfeldern der Chancen und Risiken. Pro Fragestellung werden jeweils zuerst die qualitativen Fokusgruppenergebnisse und danach die quantitativen Nachbefragungsergebnisse vorgestellt.

### Einsatzmöglichkeiten

**Figure 1:**
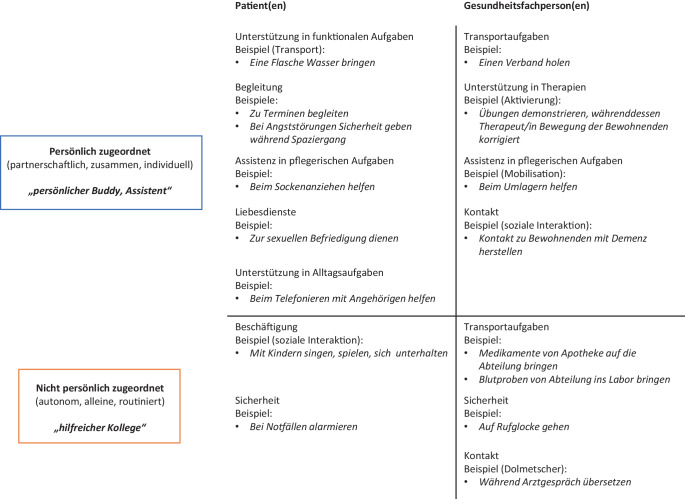

In der vertieften Analyse der Fokusgruppeninterviews zeigte sich, dass die Teilnehmenden die Einsatzmöglichkeiten sozialer Roboter unterschieden nach der Anwendergruppe der Patientinnen und Patienten (bzw. pflegebedürftige Menschen, Bewohnerinnen/Bewohner, Klientinnen/Klienten) und der Gesundheitsfachpersonen. Zusätzlich stellte sich heraus, dass die Teilnehmenden die sozialen Roboter in zwei unterschiedlichen Rollen sahen. Einerseits in einer persönlich zugeordneten Rolle als eine Art „persönlicher Buddy oder Assistent“, bei der die Mensch-Roboter-Interaktion partnerschaftlich und individuell zugeschnitten ist. Anderseits sahen die Teilnehmenden den sozialen Roboter in einer nicht persönlich zugeordneten Rolle als „hilfreicher Kollege“, bei der der Roboter autonomer und routinierter agiert. Beide Rollen des sozialen Roboters treffen dabei auf beide Anwendergruppen zu, wodurch sich die grafische Darstellung in Abb. 1 ergab.

#### Anwendergruppe Patientinnen/Patienten

In der Anwendergruppe der pflegebedürftigen Menschen fielen die meisten von den Teilnehmenden genannten Einsatzmöglichkeiten eines sozialen Roboters in die persönlich zugeordnete Rolle. Hier wurde die Rolle des „Buddy“ und Alltagsbegleiters direkt genannt, wie das nachfolgende Zitat eines Teilnehmers exemplarisch zeigt:

Ich wage die These, dass wenn soziale Roboter ausgereifter sind, wir mit ihnen zusammen den Alltag gestalten würden. Wenn ich z. B. etwas esse, sitze ich zusammen mit dem Roboter am Tisch und er kann so wie das Leben mitgestalten. (I: Gruppe C (Pflege))

Ein sozialer Roboter würde in dieser Rolle auch vermehrt Service- und Assistenzfunktionen übernehmen, wie z. B. Gegenstände bringen oder in der Körperpflege unterstützen. Auf die nicht persönlich zugeordnete Rolle fiel die Einsatzmöglichkeit der Unterhaltung, z. B. in einer Gruppe oder in einem Wartebereich. Aufgrund der autonomeren Handlungsweise des sozialen Roboters wurde das Alarmieren in einer Notfallsituation ebenfalls in dieser Kategorie eingeordnet, allerdings könnte die Rolle des sozialen Roboters in Bezug auf den unterstützungsbedürftigen Menschen auch persönlich zugeordnet sein.

#### Anwendergruppe Gesundheitsfachpersonen

In der Anwendergruppe der Gesundheitsfachpersonen sahen die Teilnehmenden die Einsatzmöglichkeiten eines sozialen Roboters in der (zumindest temporär) persönlich zugeordneten Rolle mit einer Bring-Hol-Funktion/Transportaufgabe, wie es folgender Teilnehmer äusserte: *„Wenn man im Zimmer ist und etwas vergessen hat, dass man da den Roboter schicken könnte, um es zu holen“ (I: Gruppe B (Management)). *Auch bei Mobilisationen und Positionswechsel, aber auch wenn jemand stürzt, könnten soziale Roboter eingesetzt werden. Des Weiteren könnte ein sozialer Roboter in der Unterstützung von Therapien, seien es Physio‑, Ergo‑, Logo-, oder kognitive Therapien, aber auch in der Beschäftigung eingesetzt werden. Nachfolgend wiederum ein typisches Zitat einer Pflegefachfrau:

Zur Unterhaltung sind sie sicher gut, ich stelle mir vor, wenn wir morgens das Frühstück verteilen und dann Bewohner haben, die unruhig sind oder schon wieder wegwollen, dann könnte der soziale Roboter eine Geschichte erzählen und der Bewohner würde sich so weniger alleine fühlen. (I: Gruppe D (Langzeitbereich))

In der nicht persönlich zugeordneten Rolle nannten die Teilnehmenden mehrheitlich Einsatzmöglichkeiten in Transport- und Sicherheitsaufgaben. Ein Teilnehmer beschrieb dies wie folgt: *„typisches Nachtdienstsetting im Spital, schauen, ob jemand atmet, ob sich die Decke bewegt oder ob die Person überhaupt im Bett liegt“ (I: Gruppe C (Pflege))*.

#### Nachbefragung

In der Nachbefragung gaben die Teilnehmenden für die Aufgabenbereiche des sozialen Roboters als psychologischer Therapeut, in der körperlichen Aktivierung und für pflegerische Aufgaben Zustimmungswerte für die eigenständige Durchführung im Median von 2–2,5 (IQR 1–5). Für die Durchführung zusammen mit dem medizinischen Personal lagen die Zustimmungswerte für die gleichen Aufgaben/Rollen im Median bei 4 (IQR 3–5) und damit deutlich höher. Die höchste Zustimmung gaben die Teilnehmenden dem Aufgabengebiet der Dienstleistungen in der eigenständigen Ausführung (Med = 5,5, IQR = 5–6). Dies war auch die einzige Rolle, in der die Zustimmung für das eigenständige Erledigen der Aufgabe durch einen sozialen Roboter höher bewertet wurde als dass dies zusammen mit Gesundheitspersonal geschehen soll. Die tiefsten Zustimmungswerte lagen beim eigenständigen Einsatz eines sozialen Roboters für pflegerische Aufgaben (Md = 2, IQR = 1–3). Es zeigt sich zusammenfassend eine Präferenz des sozialen Roboters bei Dienstleistungen wie z. B. dem Holen und Bringen in der Rolle „eigenständig“. Demgegenüber fand die Rolle „zusammen mit dem medizinischen Personal“ bei intensiverer Interaktion wie z. B. bei der Aktivierung oder pflegerischen Aufgaben stärkeren Anklang.

### Spannungsfelder der Chancen und Risiken

**Figure 2:**
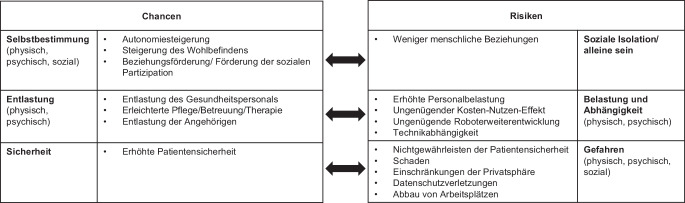

In der vertieften Analyse der Diskussion der Fokusgruppen zu den Chancen und Risiken vom Einsatz sozialer Roboter zeigte sich, dass sich die Chancen und Risiken in drei Spannungsfeldern gegenüberstellen lassen: „Selbstbestimmung vs. soziale Isolation“, „Entlastung vs. Belastung und Abhängigkeit“ und „Sicherheit vs. Gefahren“, s. Abb. 2.

#### „Selbstbestimmung vs. soziale Isolation“

Als oft genannte Chancen sahen die Teilnehmenden die Steigerung von Autonomie und Wohlbefinden sowie die Förderung von sozialer Partizipation von pflegebedürftigen Menschen durch einen sozialen Roboter. Denn ein Roboter kann auf die individuellen Vorlieben eines Menschen eingestellt werden, wodurch die Betreuung persönlich und selbstbestimmter, ohne Abhängigkeit von Gesundheitspersonal gestaltet werden kann. Eine Teilnehmerin äusserte dies folgendermassen:

Ich hatte mal eine Patientin, die mir gesagt hat, „ich hätte lieber einen Roboter als so eine Pflegefachperson, die hässig ist und es nicht so macht, wie ich es gerne will“. (I: Gruppe B (Management))

Dies kann Patientinnen und Patienten, in gewissen Situationen, von ihrem Schamgefühl *(„in manchen Bereichen, gerade beim Begleiten zur Toilette kann es ja auch schön sein, wenn nicht ein Mensch involviert ist, da ist das emotionslose der Vorteil“ (I: Gruppe B (Management)))* oder einem schlechten Gewissen (es muss keine Person um Hilfe gefragt werden) befreien. Zudem können soziale Roboter Beziehungen fördern, indem sie überhaupt Kontakt zu schwer zugänglichen Personen (z. B. Menschen mit Demenz) herstellen oder eine Person dabei unterstützen, Kontakte aufrecht zu halten (durch einfache, integrierte Kommunikationssysteme wie z. B. Telefonie).

Andererseits sahen die Teilnehmenden in einer Extremform dieser Chance auch ein Risiko. Nämlich, dass durch den vermehrten Robotereinsatz menschliche Beziehungen zu stark abnehmen könnten, dass „noch weniger Mensch-Mensch-Kontakte“ stattfänden *(„Ich finde einfach, sie können nicht menschliche Beziehungen ersetzen“ (I: Gruppe C (Pflege)))*.

#### „Entlastung vs. Belastung und Abhängigkeit“

Als weitere Chance im Einsatz von sozialen Robotern sahen die Teilnehmenden eine Entlastung des Gesundheitspersonals, der unterstützungsbedürftigen Personen selbst und der Angehörigen. Einerseits soll das Gesundheitspersonal bei repetitiven oder körperlich schweren Arbeiten unterstützt werden. Somit soll sich die körperliche Belastung und die Belastung durch Unterbrüche in der Arbeit verringern und die Teilnehmenden erhoffen sich für das Gesundheitspersonal somit mehr Zeit für direkten Patientenkontakt. Für die unterstützungsbedürftigen Personen selbst sahen die Teilnehmenden die Chance einer erleichterten Therapietreue, da es ihnen durch die Motivation durch den sozialen Roboter leichter fallen könnte, sich an die Therapie- und Medikationsempfehlungen zu halten. Wenn durch den Einsatz eines sozialen Roboters auch erreicht werden kann, dass z. B. kognitiv eingeschränkte Menschen weniger unruhig oder ängstlich sind, könnte sogar eine Medikamentenreduktion erreicht werden. Angehörige könnten insofern entlastet werden, dass ein sozialer Roboter den Gesundheitszustand von Patientinnen/Patienten überwachen könnte und so beispielweise Notfälle verhindern oder frühzeitig erkennen könnte, so sagte eine Teilnehmerin:

Eltern von schwer kranken Kindern könnten nachts vielleicht etwas schlafen, wenn sie wüssten, dass ein Roboter den Gesundheitszustand des Kindes überwacht. (I: Gruppe C (Pflege))

Auf der anderen Seite sahen die Teilnehmenden das Risiko, dass das Gesundheitspersonal aufgrund der Zusatzaufgaben in der Betreuung und Überwachung von Robotern zusätzlich belastet anstatt entlastet wird *(„Pflegende sollen keine Roboterbetreuer sein“, I: Gruppe B (Management))*. Zudem schätzten sie den Organisationsaufwand für die notwendige Umstrukturierung der Arbeitsprozesse als sehr hoch ein. Oder sie befürchteten, dass *„die Maschine überschätzt wird“ (I: Gruppe B (Management))* und in einem weiteren Schritt das Vertrauen in die eigenen Fähigkeiten sinkt. Schlussfolgernd gaben die Teilnehmenden zu bedenken, dass der Kosten-Nutzen-Effekt für die Praxis ungenügend ausfallen könnte, wie dies ein Teilnehmer formulierte: *„Entertainment gut und recht, aber wie berechne ich am Schluss den Return on Investment?“ (I: Gruppe A (Interdisziplinär)).*

#### „Sicherheit vs. Gefahren“

Durch die Möglichkeit der verbesserten Therapie, aber vor allem aufgrund der möglichen Überwachungs- und Kontrollaufgaben sahen die Teilnehmenden hohe Chancen, dass die Sicherheit von unterstützungsbedürftigen Personen erhöht wird. So sagte eine Teilnehmerin: *„Kinder von älteren Menschen wären vielleicht beruhigter, wenn sie wüssten, ein Roboter ist da“ (I: Gruppe C (Pflege))*.

Doch der erhöhten Sicherheit gegenüber sahen die Teilnehmenden auch einige Gefahren. So z. B., dass aufgrund der erhöhten Überwachung die Privatsphäre von Patientinnen und Patienten eingeschränkt ist und sich gleichzeitig das Risiko von Datenschutzverletzungen ergibt. Auch dass die Patientensicherheit in komplexen Situationen nicht mehr gewährleistet ist und Personen somit zu Schaden kommen könnten, äusserten die Teilnehmenden als Risiko. Eine Bewohnerin formulierte dies so:

Ich hätte schon Angst, dass er [der soziale Roboter] alles fallen lassen würde, oder dass wenn er mich baden würde, dass er nicht so viel Kraft hat. Ich habe da nicht so viel Vertrauen, wie wenn es ein Mensch wäre. (I: Gruppe D (Langzeitbereich))

Nicht zuletzt nannten die Teilnehmenden das Risiko, dass es zum Abbau von Arbeitsplätzen z. B. im Bereich von Pflege- oder Assistenzpersonal kommen könnte.

#### Nachbefragung

In der Nachbefragung schätzten die Teilnehmenden die Hälfte der 13 beschriebenen Chancen und vier der beschriebenen Risiken im Einsatz sozialer Roboter als eher gross ein (mit Zustimmung von über 50 % zu einer Chance resp. einem Risiko von „eher gross“), siehe Tab. 2.

**Table 2:** 

**Chance**	*Eher klein, n (%)*^*a*^	*Eher gross, n (%)*^*a*^
Unterstützung körperliche Schwerarbeit	1 (6)	15 (94)
Einfache Assistenzaufgaben	2 (13)	14 (88)
Autonomiegewinn	3 (19)	13 (81)
Unterstützung Routinearbeiten	2 (13)	13 (81)
Unterstützung Personen mit besonderen Bedürfnissen	3 (19)	13 (81)
Linderung Einsamkeit	6 (38)	10 (63)
Allgemeine Unterhaltung	6 (38)	10 (63)
Verringerung Bildschirmzeit	9 (56)	7 (44)
Förderung geistige Fähigkeiten	9 (56)	7 (44)
Förderung zwischenmenschliche Kontakte	9 (56)	7 (44)
Förderung körperliche Fähigkeiten	10 (63)	6 (38)
Kompetenzgewinn	10 (63)	6 (38)
Befriedigung sexuelle Bedürfnisse	10 (63)	5 (31)
**Risiko**	*Eher klein, n (%)*^*a*^	*Eher gross, n (%)*^*a*^
Ununterbrochene Überwachung durch Roboter	3 (19)	13 (81)
Kontaktverlust zu Menschen	4 (25)	12 (75)
Monopolstellung Herstellerfirmen	7 (44)	9 (56)
Täuschung	8 (50)	8 (50)
Entlastung Personal auf Kosten Patienten	9 (56)	7 (44)
Zusatzaufwand für Personal	9 (56)	7 (44)
Datenschutzverletzung	9 (56)	6 (38)
Verletzungsrisiko	10 (63)	6 (38)
Keinen Nutzen für Roboter finden	12 (75)	4 (25)
Kompetenzverlust	12 (75)	4 (25)
Arbeitsplatzverlust	10 (63)	5 (31)
Suchtgefahr	15 (94)	1 (6)

^a^Die Dichotomisierung der Bewertungsskala war wie folgt: Ausprägungen 1 („keine Chance“/„kein Risiko“) bis 3 = „eher klein“, Ausprägungen 4 bis 6 („grosse Chance“/„grosses Risiko“) = „eher gross“

## Diskussion

Ziel dieses Beitrags war es, die Einsatzmöglichkeiten von sozialen Robotern und die sich daraus ergebenden Chancen und Risiken bei potenziellen Anwenderinnen und Anwendern im Schweizer Gesundheitsbereich vertieft explorativ zu untersuchen. Die vertiefte Analyse fokussierte dabei auf die Beschreibung und Gewichtung der Einsatzmöglichkeiten sozialer Roboter und auf die Identifikation übergeordneter Spannungsfelder beim Einsatz sozialer Roboter im Gesundheitsbereich. Dabei zeigte sich in den Ergebnissen der Fokusgruppeninterviews, dass die Teilnehmenden die Einsatzmöglichkeiten von sozialen Robotern unterschieden in die Anwendergruppen der Patientinnen/Patienten und der Gesundheitsfachpersonen. Für beide Anwendergruppen sahen die Teilnehmenden einen sozialen Roboter entweder in einer persönlich zugeordneten oder einer nicht persönlich zugeordneten Rolle. In der Gewichtung der Einsatzmöglichkeiten wurde aus der Nachbefragung deutlich, dass die Rolle eines sozialen Roboters im Dienstleistungsbereich die höchste Zustimmung erhielt, während der Einbezug des Roboters in ein Team mit medizinischem Personal bei Aufgaben der Pflege und der Aktivierung präferiert wurde. Bei diesen Aufgaben sehen es die Teilnehmenden offensichtlich nicht, dass pflegebedürftige Menschen allein von einem Roboter betreut werden, sondern von einem Team bestehend aus medizinischem Personal und Roboter. Die Chancen und Risiken sozialer Roboter, die sich aus den Einsatzmöglichkeiten ergeben, liessen sich in der vertieften Analyse in die drei Spannungsfelder „Selbstbestimmung vs. soziale Isolation“, „Entlastung vs. Belastung & Abhängigkeit“ und „Sicherheit vs. Gefahren“ einteilen. Die grössten Chancen lagen, gemäß der Nachbefragung, in den Bereichen „Selbstbestimmung“ und „Entlastung“, die grössten Risiken sahen die Teilnehmenden in den Bereichen „Gefahren“ und „soziale Isolation“.

Die sich in diesem Beitrag erstmalig gezeigte Unterscheidung der Einsatzbereiche sozialer Roboter für die Anwendergruppen der pflegebedürftigen Menschen und des Gesundheitspersonals verdeutlicht den Nutzen, den der Einsatz eines sozialen Roboters für die verschiedenen Anwendergruppen im Gesundheitsbereich hat. In der verfügbaren Literatur wurde bisher vor allem nach der Art des Roboters unterschieden (z. B. Assistenzroboter, Telepräsenzroboter, Therapieroboter, sozialer Roboter) (Becker et al. 2013). Die in diesem Beitrag dargestellte Unterscheidung in der persönlich zugeordneten und nicht persönlich zugeordneten Rolle eines sozialen Roboters unterstreicht die Bedeutsamkeit der Mensch-Roboter-Beziehung. Sie lässt sich in der Taxonomie der Mensch-Roboter-Interaktion in der Teamklassifikation von Onnasch und Roesler (2021) wiederfinden. Die menschliche Rolle des „Zusammenarbeiters“ („collaborator“) lässt sich gut übertragen auf die in diesem Beitrag genannte Roboterrolle des „persönlichen Buddy/Assistenten“, in der die Zusammenarbeit mit dem sozialen Roboter als partnerschaftlich beschrieben wird und die Rolle des sozialen Roboters womöglich als eher aktiv wahrgenommen wird. Die menschliche Rolle des „Mitarbeiters“ („cooperator“) (Onnasch und Roesler 2021) kann in der Roboterrolle des „hilfreichen Kollegen“ wiedergefunden werden.

Beans (2018) betont in diesem Zusammenhang allerdings, dass eine wirkliche Zusammenarbeit mit Robotern heutzutage noch nicht möglich sei, da diesen ein Situationsbewusstsein fehle, und sie dadurch nicht in der Lage seien, zu verstehen, wie sich die Vielfalt und Nuancierung des menschlichen Verhaltens in verschiedenen Kontexten verändert. Die aktuell noch nicht mögliche Form einer wirklichen Zusammenarbeit erklärt wahrscheinlich auch, warum die Teilnehmenden dem Dienstleistungsbereich, also z. B. dem Holen und Bringen von Gegenständen, eine hohe Chance als Einsatzmöglichkeit für soziale Roboter zugesprochen haben. Gemäß den von den Teilnehmenden geäusserten Einsatzmöglichkeiten enthält der soziale Roboter mehr funktionale als soziale Funktionen, welche eher denen eines Serviceroboters entsprechen und für den Dienstleistungsbereich relevant sind. Dennoch sahen die Teilnehmenden diesen „Dienstleistungsroboter“ als sozialen Roboter an und nicht als nicht-sozialen Assistenzroboter. Sie verdeutlichten damit, dass die Vermischung der Funktionen einem Bedürfnis der potenziellen Anwenderinnen und Anwender entspricht. Daher passt die Aufgliederung von Busse et al. (2021), in der ein Serviceroboter zu den sozialen Robotern gehört, sehr gut zu den Vorstellungen der Teilnehmenden. Es hebt hervor, dass die zukünftige Roboterentwicklung sich nicht zu starr auf die Trennung von sozialen Robotern und Servicerobotern fokussieren sollte, sondern die optimale Abstimmung zwischen funktionalen und sozialen Funktionen gesucht werden soll. In anderen Studien, in denen z. B. Pflegepersonen gefragt wurden, welche Bedürfnisse sie an einen Roboter als Unterstützung in der Pflege haben, wurden die Bereiche Überwachung, Mobilität und Sicherheit genannt, allerdings wurde der Dienstleistungsbereich im Sinne des Holens und Bringens von Gegenständen und des Assistenten für Pflegefachpersonen bisher noch kaum untersucht (Lee et al. 2020).

Als Einsatzmöglichkeiten von sozialen Robotern für pflegebedürftige Menschen werden in der Literatur häufig stark eingeschränkte Menschen, z. B. Menschen mit Demenz oder ausgeprägter Autismusspektrumstörung genannt. Diese Menschen, wie auch Kinder, zählen zu den vulnerablen Patientengruppen. Forschung zum Einsatz sozialer Roboter bei weniger vulnerablen Gruppen, also nicht oder nur wenig eingeschränkten Personen, ist noch wenig vorhanden (Zöllick et al. 2021). Die teilnehmenden Gesundheitsfachpersonen des vorliegenden Projektes sahen, bezogen auf diese Personengruppe mit weniger gesundheitlichen Einschränkungen, am ehesten den Einsatz eines sozialen Roboters als persönlichen Buddy/Alltagsbegleiter. Auch andere Untersuchungen zeigten, dass soziale Roboter vor allem als Begleiter in sozialen Situationen wie einem gemeinsamen Essen, beim Fernsehen zuhause oder als Begleiter bei einem Spaziergang gewünscht sind (Chen et al. 2019; Karunarathne et al. 2019).

In der Nachbefragung schätzten die Teilnehmenden als die zwei grössten Risiken den Verlust der Privatsphäre, durch eine erhöhte oder ununterbrochene Überwachung von pflegebedürftigen Menschen durch soziale Roboter bzw. letztendlich durch Angehörige oder Gesundheitsfachpersonen, und den menschlichen Kontaktverlust ein. Auch im Review von Zöllick et al. (2021) wurde das Risiko des Privatsphärenverlusts genannt. Daher scheint es wichtig, dass ein Aushandlungsprozess über die Art und Häufigkeit der Überwachung durch soziale Roboter zwischen Betroffenen und Gesundheitspersonal vorangegangen sein muss, damit die Akzeptanz des Roboters gewährleistet ist. Auch in Bezug auf das Risiko des menschlichen Kontaktverlustes wird immer wieder betont, dass der menschliche Kontakt und die Pflege nicht ersetzt, sondern unterstützt oder ergänzt werden soll (Rebitschek und Wagner 2020; Zöllick et al. 2021). Um diese Forderung zu verwirklichen, scheint es wichtig, dass vor dem Einsatz eines sozialen Roboters dessen Aufgabenbereich definiert und gezielt festgelegt wird. Zudem sollten betroffene Personen stets wählen können, ob sie durch einen Roboter oder durch einen Menschen gepflegt oder betreut werden möchten. Das Recht auf menschliche Pflege ist hierbei zu gewährleisten, vergleiche Suwa et al. (2020). Denn gemäß Rebitschek und Wagner (2020) lehnt rund ein Drittel der Erwachsenen die Assistenz durch Roboter grundsätzlich ab. Die vier in diesem Projekt in der Nachbefragung genannten höchsten Risiken eines Robotereinsatzes (mit über 50 % Zustimmung) betreffen die in den Fokusgruppeninterviews genannten Bereiche „soziale Isolation“ und „Gefahren“. Das Risiko des Arbeitsplatzverlustes wurde in den Fokusgruppeninterviews zwar genannt und auch in der vorhandenen Evidenz als ein potenzielles Risiko beschrieben (Beans 2018), in der Nachbefragung wurde dieses jedoch als gering eingeschätzt. Zusätzlich wurden in den Fokusgruppeninterviews die Risiken des ungenügenden Kosten-Nutzen-Effekts, der Technikabhängigkeit und des potenziellen Nichtgewährleistens der Patientensicherheit genannt, welche als Themen nicht in der Nachbefragung enthalten waren und daher keine Quantifizierung durch die Teilnehmenden zuliessen.

Gegenüber den Risiken finden sich in der Nachbefragung die am höchsten bewerteten Chancen (mit über 50 % Zustimmung) in den Bereichen der „Entlastung“ – hier eine physische Entlastung – und der „Selbstbestimmung“ von pflegebedürftigen Personen. Diese Chancen decken sich durchaus mit der vorhandenen Evidenz (Becker et al. 2013; Zöllick et al. 2021). Allerdings wurden in den Fokusgruppeninterviews auch oft die Erhöhung der Sicherheit für pflegebedürftige Personen genannt und somit eine mögliche psychische oder mentale Entlastung der Angehörigen. Diese Aspekte wurden bisher nur wenig untersucht, es lassen sich allerdings Parallelen finden zu Wang et al. (2017). Dort äusserten die Angehörigen von Menschen mit Demenz, dass durch einen Robotereinsatz Frustrationen, Stress und Beziehungsstress gemindert würden.

Eine der grössten Stärken dieses Projektes war, dass die Perspektiven von potenziellen Anwenderinnen und Anwendern von sozialen Robotern im Gesundheitsbereich in der Schweiz untersucht wurden, wodurch die Evidenz für Robotereinsätze im Schweizerischen Gesundheitsbereich zu den Ergebnissen von Busse et al. (2021) erweitert wurde. Zudem war es wichtig, dass nicht nur eine Berufsgruppe (z. B. Pflegefachpersonen) als potenzielle Anwendergruppe in die Datensammlung eingeschlossen wurde, sondern dass auch andere Gesundheitsfachpersonen und Experten aus dem Gesundheitswesen (z. B. IT) und v. a. Bewohnende eines Pflegeheims am Projekt teilgenommen haben. Dies unterstützt den in der Literatur geforderten frühzeitigen Einbezug aller möglichen Anwendergruppen für die erfolgreiche Implementierung sozialer Roboter (Papadopoulos et al. 2020). Die in diesem Beitrag erstellte Darstellung der Einsätze eines sozialen Roboters nach Anwendergruppen und in eine persönlich zugeordnete und nicht-persönlich zugeordnete Rolle des Roboters ist nach dem Kenntnisstand der Autorinnen und Autoren erstmalig so erfolgt. Diese Darstellung vereinfacht die wichtige Aufgabe, vor dem Einsatz eines sozialen Roboters eine klare Aufgabendefinition zu beschreiben und einen möglichen Einsatz zielgerichtet und bedürfnisorientiert zu gestalten. Dies sollte dazu beitragen, das Kosten-Nutzen-Verhältnis für den Einsatz eines sozialen Roboters ausgewogen zu gestalten. Als weitere Stärken können die Roboterszenarien, insbesondere die Live-Szenarien, genannt werden. Da die Teilnehmenden mehrheitlich noch wenig Praxiserfahrung mit sozialen Robotern hatten, ermöglichten ihnen die Roboterszenarien eine realistischere Einschätzung der Einsatzmöglichkeiten, Chancen und Risiken. Des Weiteren erwies sich die Datensammlungs- und Analysemethode des Knowledge Mappings als geeignet, um die Forschungsfragen ressourcenschonend zu beantworten und den Teilnehmenden die ersten Ergebnisse sofort zu präsentieren.

Limitierend für das Projekt war, dass die Fragebogenentwicklung für die Nachbefragung vor der Durchführung des Round Tables stattfand, wodurch nicht alle in den Fokusgruppeninterviews genannten Themen integriert werden konnten. Dadurch war eine Quantifizierung der Ergebnisse aus den Interviews nicht in allen Bereichen möglich. Zudem war es aus Ressourcengründen nicht möglich, dass die Bewohnenden an der Nachbefragung teilnahmen, wodurch die Aussagekraft etwas eingeschränkt ist. Zudem limitierte die COVID-19-Pandemie das Projekt, da dadurch der Round Table verschoben werden musste und bereits rekrutierte Teilnehmende nicht mehr teilnehmen konnten. Bedauerlicherweise betraf dies hauptsächlich ärztliche und therapeutische Fachpersonen, wodurch diese potenziellen Anwendergruppen untervertreten waren. Die allermeisten Beteiligten aus der daraus resultierenden übervertretenden Gruppe des Managements waren jedoch beruflich im Gesundheitsbereich sozialisiert, wodurch diese Limitation zumindest anteilig kompensiert werden konnte. Auch die Rekrutierung von Patientinnen/Patienten (z. B. aus Akutspitälern) und Klientinnen/Klienten aus der Spitex und somit von Erwachsenen und Personen mit weniger starken gesundheitlichen Einschränkungen, war pandemiebedingt leider nicht erfolgreich. Allerdings zeigte sich, dass die Teilnehmenden eine hohe Reflexionsfähigkeit aufwiesen, wodurch diese Perspektiven dennoch zu Teilen abgebildet werden konnten. Für die weitere Verifizierung dieser ersten Ergebnisse zu diesen Personengruppen sind weitere Studien erforderlich, in denen die Betroffenen selbst befragt werden.

## Fazit

Mit dieser Untersuchung konnte gezeigt werden, dass potenzielle Anwenderinnen und Anwender von sozialen Robotern im schweizerischen Gesundheitsbereich einige Einsatzmöglichkeiten und Potenziale für soziale Roboter in ihrem Anwendungsfeld sehen. Aus Sicht der Teilnehmenden ist ein sozialer Roboter in der Praxis am ehesten einsetzbar im Dienstleistungsbereich, z. B. in Form eines persönlichen Buddys für pflegebedürftige Menschen oder Gesundheitsfachpersonen, der Dinge holt und bringt oder in Form eines hilfreichen Kollegen, der, eingebunden in ein Pflegeteam, Transporte erledigt, z. B. Apothekenlieferungen oder Getränkebestellungen. Bei intensiverer Interaktion, wie z. B. bei pflegerischen Tätigkeiten, ist unbedingt zu beachten, dass diese, wenn überhaupt, zusammen mit medizinischem Personal geschieht. Zudem zeigen die Ergebnisse deutlich, dass die potenziellen Anwenderinnen und Anwender im Verlust menschlicher Kontakte und in der Fokussierung auf Betreuung durch Roboter auch hohe Risiken sahen. Diese Risiken müssen möglichst minimiert werden, wenn in der Praxis überlegt wird, einen sozialen Roboter zu implementieren. Dazu ist es wichtig, dass die zukünftigen Anwenderinnen und Anwender von Anfang an einbezogen werden, damit die Akzeptanz des sozialen Roboters auch gegeben ist und die Organisationsstrukturen erfolgsversprechend angepasst werden können. Es ist wichtig, die möglichen Einsatzbereiche eines sozialen Roboters für verschiedene Personengruppen zu definieren und festzulegen, und sich hierbei nicht nur auf die vulnerabelsten Personengruppen, wie z. B. stark funktionseingeschränkte oder kognitiv eingeschränkte Menschen zu beschränken. Denn die Teilnehmenden sahen auch bei weniger eingeschränkten Personengruppen ein grosses Potenzial für den Einsatz sozialer Roboter.
